# Simultaneous Determination of Malachite Green, Chloramphenicols, Sulfonamides, and Fluoroquinolones Residues in Fish by Liquid Chromatography-Mass Spectrometry

**DOI:** 10.1155/2020/3725618

**Published:** 2020-02-19

**Authors:** Yongping Chen, Sudong Xia, Xianqin Han, Zhiru Fu

**Affiliations:** ^1^Tianjin Agricultural Ecological Environment Monitoring and Agricultural Product Quality Testing Center, Tianjin 300221, China; ^2^Tianjin Key Lab of Aqua-Ecology and Aquaculture, Department of Fishery Science, Tianjin Agricultural University, Tianjin 300384, China

## Abstract

A fast-analytical method using simplified extraction has been developed for the simultaneous determination of 42 compounds from 4 different classes of veterinary drugs (amphenicols, triphenylmethane, fluoroquinolones, and sulfonamides) in fish by reverse phase liquid chromatography-tandem mass spectrometry. The selection of extraction reagents was optimized using different types of microfiltration membrane, mobile phase, and LC column. Samples were extracted using 0.4% hydrochloric acid in acetonitrile and ethyl acetate and then were cleaned up using solid-phase extraction Cleanert Alumina N columns (500 mg) and Oasis hydrophilic-lipophilic balance (HLB) cartridges. The chromatographic separation was performed on a XR-ODS C_8_ column using a mobile phase of (A) 0.1% formic acid and 2 mM ammonium acetate and (B) 0.1% formic acid acetonitrile at a flow rate of 0.25 mL·min^−1^. The results indicated 67.7–112.8% recovery of 42 compounds with an intra- and interday relative standard deviations less than 10%. The limits of quantification for analytes were in the range of 0.3–1.0 *μ*g kg^−1^ for samples which were satisfactory to support future surveillance monitoring. The method applicability was checked by analyzing 30 fish samples collected from local markets. Two fish samples surpassed the established MRL of 100 *μ*g kg^−1^ with values of 104 *μ*g kg^−1^ and 112 *μ*g kg^−1^.

## 1. Introduction

Antibiotics are used widely to treat animal diseases in aquacultures, such as bacterial infectious diseases, and as feed additives to promote the growth of aquaculture animals [1–3]. Beyond that, antibiotics are used extensively in humans and animals to prevent and treat diseases. A large number of antibiotics that have not been metabolized can enter the aquatic environment through direct discharge of animal wastewater or by leaking or running off from manure fertilizer on agricultural land [4]. Eventually, antibiotics can be present in the water and sediment of the living environment of aquaculture animals. Antibiotics in the environment can lead to residuals in aquatic animals [5, 6]. Chloramphenicol, florfenicol, malachite green, sulfonamides (SAs), and quinolones (QNs) are effective topical fungicides used in the aquaculture industry [7]. Chloramphenicol, florfenicol, and malachite green are prohibited as aquaculture veterinary drugs in many countries including the United States, Canada, and China and in the European Union. However, these drug residues which settle in aquatic tissues can cause some adverse human health effects, such as allergic reactions and alterations in the delicate balance of intestinal flora, and they can produce resistance to antibiotics [8–14]. Malachite green and its metabolites are toxic, mutagenic, and carcinogenic. A number of studies have been described for the determination of antibiotics, malachite green, and leucomalachite green in different sample treatment methods and instrumental determination methods [15–18]. However, these methods are focused only on single-drug class of veterinary residues, which leads to low efficiency and high cost when different physicochemical properties of various veterinary drugs are determined separately. The use of multiresidue methods for the simultaneous determination of different classes of compounds is a widely spreading form of analysis in laboratories that perform official controls, research facilities, or laboratories in the private sector [19]. The use of multiresidue methods is becoming necessary in routine laboratories for the analysis of veterinary drugs (e.g., SAs, QNs, and tetracyclines). Therefore, the research trend is to develop methods which can determine multiclass compounds in a single analytical process by liquid chromatography-tandem mass spectrometry (LC-MS/MS).

The development of simultaneous multiclass drug residue determination is challenging work because concentrations of analyte are low in the tissues of the fish. In addition, there is the inherent complexity of matrices containing high protein and fat content [20]. The analytical procedure normally includes extraction, purification, and instrumental determination of compounds. Extraction and purification are crucial steps to achieve the satisfactory recovery and purifying effect simultaneously for different classes of compounds from fish sample. There are many studies on the determination of sulfonamides (SAs) [21–24], fluoroquinolones (QNs) [25–31], malachite green, and leucomalachite green and their application in food matrices or environmental matrices [32–34]. It is inefficient not to meet the requirement of determining different kinds of compounds in fish sample simultaneously. It is necessary to increase the efficiency and reduce the cost and time of these analyses.

The objective of the present study is to develop and validate an analytical method using LC-MS/MS to simultaneously extract and analyze amphenicols, leucomalachite green, malachite green, QNs, and SAs in fish sample. Analytical performance of the proposed method was evaluated through a validation study which involved assessment of parameters including linearity, specificity, recovery, precision, and limits of detection (LODs) and limits of quantification (LOQs). The developed method was applied to determine the concentration levels of the selected SAs, QNs, chloramphenicol, florfenicol, leucomalachite green, and malachite green in fish sample.

## 2. Materials and Methods

### 2.1. Materials and Reagents

All solvents were of MS grade. Acetonitrile and ethyl acetate were supplied from Merck (Darmstadt, Germany). Formic acid was purchased from Sigma-Aldrich (Saint Louis, MO, USA). Hydrochloric acid was provided by Kemiou (Tianjin, China). Ammonium acetate was obtained from Sigma-Aldrich. Water was purified using a Milli-Q Synthesis system from Millipore (Bedford, MA, USA). SPE Cleanert Alumina N columns (500 mg) were provided by Agela (Tianjin, China). Oasis HLB cartridges (6 mL, 200 mg or 6 mL, 500 mg) were supplied by Waters (Milford, MA, USA). Syringe filters (GHP ACRODISC 13 0.2 *μ*m) were purchased from Pall Corporation (Ann Arbor, MI, USA).

The target standards of sulfisoxazole, sulfisomidine, sulfathiazole, sulfapyridine, sulfamonomethoxine, sulfamethoxypyridazine, sulfamethoxazole, sulfamethizole, sulfamethazine, sulfameter, sulfamerazine, sulfaguanidine, sulfadoxine, sulfadimethoxine, sulfadiazine, sulfachloropyridazine, sulfachinoxalin, sulfabenzamide, sulfachloropyrazine, sulfaphenazole, sulfacetamide, sulfamoxol, fleroxacin, ofloxacin, norfloxacin, enoxacin, ciprofloxacin, enrofloxacin, lomefloxacin, danofloxacin, orbifloxacin, difloxacin, sarafloxacin, sparfloxacin, oxolinic acid, flumequine, pefloxacin, nalidixic acid, chloramphenicol, florfenicol, leucomalachite green and malachite green, sulfadoxine-D3, sulfadimethoxine-D6, norfloxacin-D5, ciprofloxacin-D8, enrofloxacin-D5, chloramphenicol-D5, malachite green-D5, and leucomalachite green-D6 were purchased from Dr. Ehrenstorfer, GmbH (Augsburg, Germany).

About 10 mg of individual standard (corrected by purity and salt form) was accurately weighed. The compounds were dissolved in 10 mL of acetonitrile. Standard stock solutions (1 mg mL^−1^) were stored at −20°C and were stable for at least 6 months. Mixed working standard solutions were prepared by diluting the stock solution with acetonitrile. These solutions were stored at 4°C and were stable for 3 months.

The ammonium acetate solution was prepared by dissolving 0.08 g of CH_3_COONH_4_ in a 500 mL volumetric flask with about 500 mL of water and aqueous 0.1% formic acid. Acidified acetonitrile was prepared by mixing 4 mL of hydrochloric acid and 1 L acetonitrile. HLB cartridges were prepared by activating with 6 mL methanol and 6 mL formic acid-water solution (pH = 3).

### 2.2. Extraction and Cleanup of Veterinary Drug Residue

For the extraction of compounds in fish samples which were obtained from an aquafarm, previously homogenized tissue was weighed (5.0 ± 0.02 g) into a 50 mL polypropylene tube, and 100 *μ*L of mixed internal standard working solutions was added to samples (sulfadoxine-D3, sulfadimethoxine-D6, norfloxacin-D5, ciprofloxacin-D8, enrofloxacin-D5 1.0 *μ*g mL^−1^, chloramphenicol-D5, malachite green-D5, and leucomalachite green-D6 40 ng mL^−1^). 10 mL ethyl acetate was added to each tube, and then the suspension was homogenized with a T18 basic Ultra Turrax for 2 min. The suspension was sonicated for 25 min in water bath at 35°C and then centrifuged (Sigma 3-18KS, Osterode am Harz, Germany) at 3743 ×*g* for 10 min at 4°C. The supernatant was transferred to a 20 mL glass centrifuge tube. Anhydrous sodium sulfate (10 g) was added to the residue and then 10 mL acidified acetonitrile was added to the residue followed by mixing on a vortex mixer for 2 min. All polypropylene tubes were then ultrasonicated for 15 min and centrifuged at 10,397 ×*g* for 10 min at 4°C. The supernatant from each tube was transferred to a 20 mL glass centrifuge tube combined with the first extraction. The extracted solution from each sample was dried in a water bath at 40°C under nitrogen to remove the organic solvent and then dissolved with 5 mL water. Dissolved sample extract was adjusted to pH 3 [35] using 4 M H_2_SO_4._

Sample extracts contained a lot of fat and protein, which could cause matrix interference. SPE Cleanert Alumina N columns (500 mg) and HLB cartridges (6 mL, 200 mg) were set up for cleaning up and enriching the aqueous solutions of sample extracts. SPE Cleanert Alumina N column was placed on top of the HLB cartridge for removing polar impurities. Five mL of sample extract was passed through the cartridge at a flow rate of 5 mL·min^−1^. Then, the SPE Cleanert Alumina N column was removed, and the HLB cartridge was rinsed with 5 mL water to remove weakly bound impurities. The analytes were eluted with 8 mL 0.01% formic acid-methanol, and then the eluates were evaporated to dryness under a gentle stream of nitrogen at 35°C. The dried extract was reconstituted in 2 mL of 10% acetonitrile solution. The reconstituted solution was mixed on a vortex mixer for 2 min and then 1 mL reconstituted solution was transferred to a 2 mL centrifuge tube and centrifuged (Eppendof 5424, Hamburg, Germany) at 12,638 ×*g* for 10 min at 4°C. The supernatant was filtered through a 0.2 *μ*m syringe filter (GHP ACRODISC 13 0.2 *μ*m) into a glass LC vial for analysis by LC-MS/MS.

### 2.3. Instrumental Conditions

In order to achieve better sensitivity and selectivity, the MS parameters were optimized by infusing a standard solution of 0.2 *μ*g mL^−1^ of each analyte. Collision energies were optimized in order to find the most abundant product ions. The work monitored two fragments, selecting the most intensive transition for quantification and another for confirmation. The optimization of the ESI source temperature, curtain gas, ion spray voltage, Gas1, and Gas2 in positive and negative mode by flow injection analysis (FIA) has increased the method's sensitivity and the ionization's efficiency. The optimization of MS parameters carried out in the MS/MS and the optimization of the ESI in positive and negative mode are shown in Table 1.

The target compounds were analyzed by LC-MS/MS (SHIMADZU liquid chromatography LC-30AD system coupled to an AB 5500 Qtrap triple quadrupole mass spectrometer) in multiple-reaction monitoring (MRM) mode. The analyses were performed in the negative mode for two target compounds (CAP and FF) and in the positive mode for the other compounds. Nitrogen was used as the collision gas.  Table 2 shows LC and MS parameters. Mass spectrometric conditions were optimized using an optimizer (AB, Palo Alto, CA, USA) for collision energy (CE) and declustering potential (DP).  Table 2 shows MS/MS transitions for quantification and confirmation as well as CE and DP values optimized for each of the selected compounds.

Different kinds of LC columns were tested for separation of compounds. When a Thermo C_18_ column (100 × 2.1 mm, 5 *μ*m) was used for all the compounds with the mobile phase of A: 0.1% formic acid with 2 mM ammonium acetate and B: 0.1% formic acid in acetonitrile, some compounds could not produce a conclusive peak shape and sensitivity and could not be separated by chromatography. By using a CAPCELL PAK C_18_ MG II column (100 × 2.0 mm, 5 *μ*m) for all the compounds with the mobile phase of A: 0.1% formic acid with 2 mM ammonium acetate and B: 0.1% formic acid in acetonitrile, although some compounds could be separated by chromatography, other compounds could not produce a conclusive peak shape, whereas Shim-pack XR-ODS (75 mm × 2.1 mm, 2.2 *μ*m) gave a narrow peak, better sensitivity, and calibration curve linearity for each analyte compared to the two other columns. Based on the comparative experiment, Shim-pack XR-ODS (75 mm × 2.1 mm, 2.2 *μ*m) was chosen for the best separation of all target compounds.

When 42 compounds are analyzed simultaneously and the chemical structures of the analytes differ greatly and the optimization of chromatographic separation is very difficult. There are fifteen analytes, oxolinic acid, olumequin, oulfaguanidine, sulfacetamide, sulfamethoxazole, sulfisoxazole, sulfamethazine, sulfisomidine, sulfameter, sulfamethoxypyridazine, sulfamonomethoxine, sulfachloropyridazine, sulfachloropyrazine, sulfadoxine, and sulfadimethoxine, that have to be separated by chromatography due to the same transitions using Shim-pack XR-ODS (75 mm × 2.1 mm, 2.2 *μ*m) LC column. During method optimization, the mobile phase compositions tested were (1) A: 0.1% formic acid in water, B: 0.1% formic acid in methanol, (2) A: 0.1% formic acid with 2 mM ammonium acetate, B: 0.1% formic acid in methanol, (3) A: 0.1% formic acid with 5 mM ammonium acetate, B: 0.1% formic acid in methanol, (4) A: 0.1% formic acid in water, B: 0.1% formic acid in acetonitrile, (5) A: 0.1% formic acid with 2 mM ammonium acetate, B: 0.1% formic acid in acetonitrile, and (6) A: 0.1% formic acid with 5 mM ammonium acetate, B: 0.1% formic acid in acetonitrile. The mobile phase with A: 0.1% formic acid with 2 mM ammonium acetate and B: 0.1% formic acid in acetonitrile can achieve a better chromatographic resolution and peak sensitivity for all compounds compared to using other mobile phases.

### 2.4. Method Validation

#### 2.4.1. Linearity

The linearity was evaluated by standard addition calibration curves at different spiked levels. Calibration curves were obtained for Grass Carp matrix with concentrations based on the response of each analyte. The compounds were categorized into three groups: group 1 included sulfisomidine, sulfathiazole, sulfapyridine, sulfamonomethoxine, sulfamethoxypyridazine, sulfamethoxazole, sulfamethizole, sulfamethazine, sulfameter, sulfaguanidine, sulfadoxine, sulfadimethoxine, sulfadiazine, sulfachloropyridazine, sulfachinoxalin, sulfabenzamide, sulfaphenazole, sulfacetamide, sulfamoxol, fleroxacin, ofloxacin, enoxacin, enrofloxacin, danofloxacin, orbifloxacin, difloxacin, oxolinic acid, flumequine, pefloxacin, nalidixic acid, leucomalachite green, and malachite green, with the following spiking levels: 0.4, 1, 2, 5, 10, 20, and 50 *μ*g kg^−1^. Group 2 included sulfisoxazole, sulfamerazine, sulfachloropyrazine, norfloxacin, ciprofloxacin, lomefloxacin, sarafloxacin, and sparfloxacin, with the following spiking levels: 1, 2, 5, 10, 20, 50, and 100 *μ*g kg^−1^. Group 3 included chloramphenicol and florfenicol with the following spiking levels: 0.5, 1.0, 2, 5, 10, and 20 *μ*g kg^−1^.

#### 2.4.2. Accuracy and Precision

The recovery was used to evaluate the accuracy of the method. Six replicates of spiked samples at three concentration levels were prepared. The concentrations of spiked samples were calculated by calibration curves. The recovery was determined by means of the measured concentration. The precision, expressed as relative standard deviation (RSD), was determined by intra- and interday assays.

#### 2.4.3. Limit of Detection and Limit of Quantification

The LOD and LOQ were determined at a signal-to-noise ratio (S/N) of about 3 and 10, respectively.

*(1) Qualitative Analysis*. Positive identification of compounds in fish samples was based on the criteria for retention time (deviation within 5%). The ion ratio deviation of analytes and the standard should accord with Table 3. The method was satisfactory in terms of linearity, recovery, precision, and analytical limits under the requirements of the US FDA criteria.

## 3. Results and Discussion

### 3.1. Optimization of Extraction for Samples

**Figure 1:**
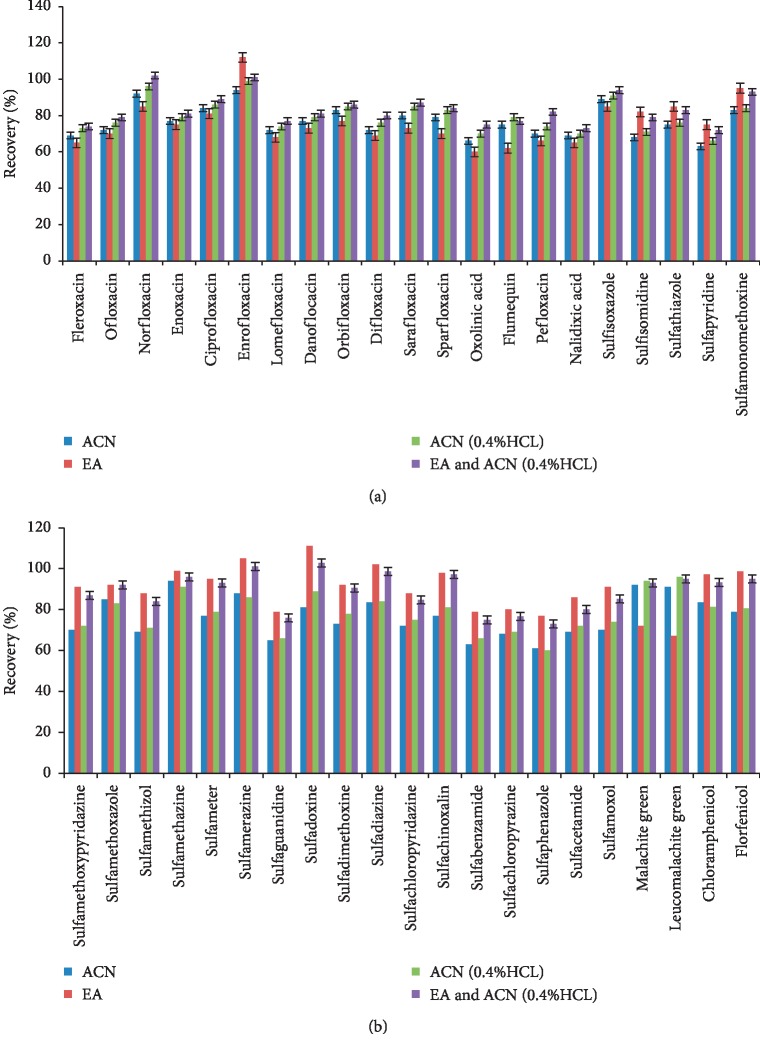
Effect of different 439 extraction solvents on recovery (%) of 42 compounds.

In order to perform the simultaneous detection of SAs, QNs, chloramphenicol, florfenicol, leucomalachite green, and malachite green, the extraction method must be capable of effectively extracting each compound from the complex fish sample matrix. The extraction procedure is a critical step because it must be able to perform a good recovery of several compounds with different chemical properties. To optimize the extraction procedure, four different solvents, acetonitrile, ethyl acetate, 0.4% hydrochloric acid in acetonitrile, and ethyl acetate followed by 0.4% hydrochloric acid in acetonitrile, were evaluated as extraction solvents.  Figure 1 shows that an acceptable recovery of compounds was only obtained by the two-step extraction utilizing ethyl acetate followed by 0.4% hydrochloric acid in acetonitrile. The use of acetonitrile and acidified acetonitrile as extraction solvents provided a better recovery of QNs, leucomalachite green, and malachite green, compared to SAs. However, the extraction efficiency of acidified acetonitrile was higher than that of acetonitrile. Using ethyl acetate as extraction solvents provided a better recovery of SAs, chloramphenicol, and florfenicol than QNs, leucomalachite green, and malachite green. The test results showed that acidified acetonitrile and ethyl acetate are suitable for extraction of all compounds because it provided the most satisfactory recoveries from the spiked samples.

Different kinds of extraction methods using a vortex mixer, ultrasonic bath, and homogenizer were tested for this experiment. In this work, extraction efficiency using the vortex was low. The ultrasonic bath yielded high extraction efficiency, which may be related to the state of the pulverized matrix, but poor stability. The more homogeneous and dispersed the matrix, the higher the extraction efficiency. The homogenizer provided higher stability, but poor extraction efficiency. Based on the above comparison, homogenization followed by ultrasonic extraction was selected as the optimized extraction procedure. Further optimization of ultrasonic treatment time was carried out by sonicating for 5, 10, 15, 20, 25, 30, and 35 minutes with an ultrasonic bath. The experimental results showed that recovery increased with longer sonication time; the recovery reached a peak at 25 minutes and tended to be stable. Ultrasonic treatment for 25 minutes was chosen. Five different kinds of microfiltration membranes SHIMADZU-GL II MCE 0.22 *μ*m, GHP ACRODISC 13 0.2 *μ*m, nylon 0.22 *μ*m, AGILENT PTFE 0.2 *μ*m, and Waters ACRODIC Syringe 0.2 *μ*m GHP were tested. When the sample extract went through a 0.22 *μ*m nylon membrane, the constituents in the microfiltration membrane were dissolved, which caused interference to QNs. SHIMADZU-GL II MCE 0.22 μm, AGILENT PTFE 0.2 *μ*m, and Waters ACRODIC Syringe 0.2 *μ*m could not remove fat and protein successfully. Only the GHP ACRODISC 13 0.2 *μ*m membrane was suitable for filtration.

### 3.2. Method Validation

#### 3.2.1. Linearity

Standard calibration curves were performed to achieve good accuracy and to compensate for the matrix effect and loss in the sample preparation. The calibration curves for analytes were constructed by plotting the ratio of analyte peak area to internal standard peak area in response of compounds (*y*) versus concentration (*x*) of each analyte which was expressed by the equation. The calibration curves were generated daily from the peak area responses of standards with concentrations ranging from 5 to 150 ng mL^−1^. Good linearity was found in the studied ranges with coefficients of determination (*R*^2^ ≥ 0.99).

#### 3.2.2. Limit of Detection (LOD) and Limit of Quantification (LOQ)

The negative samples were selected and spiked with the standard solution and then treated and analyzed following the method described above. The LODs and LOQs for each analyte were obtained from a signal-to-noise ratio (S/N) of about 3 and 10 in Table 4. The LODs for norfloxacin, ciprofloxacin, lomefloxacin, sarafloxacin, sparfloxacin, sulfachloropyrazine, sulfisoxazole, and sulfisomidine were 0.25 *μ*g kg^−1^ and the LOQs were 1.0 *μ*g kg^−1^. The LODs for CAP and FF were 0.15 *μ*g·kg^−1^ and the LOQs were 0.5 *μ*g kg^−1^. The LODs for other analytes were 0.1 *μ*g kg^−1^ and the LOQs were 0.4 *μ*g kg^−1^.

#### 3.2.3. Accuracy and Precision

**Figure 2:**
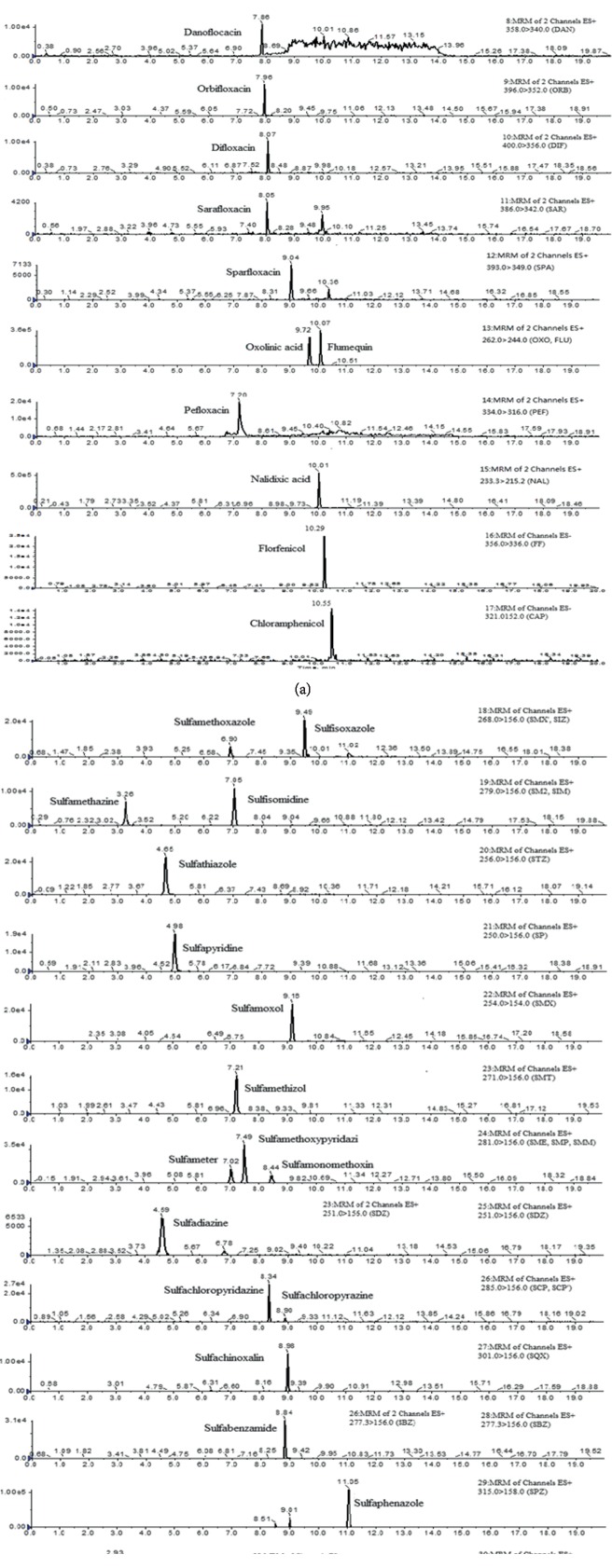
UPLC-MS/MS of 455 chromatograms of spiked fish muscle sample at LOQ level.

All recoveries are between 70% and 120%, except for oxolinic acid, flumequine, sulfapyridine, nalidixic acid, and sulfachinoxalin which show recoveries lower than 70% at some spiked levels, but the overall recovery is still acceptable. The recoveries shown in Table 5 are between 67.7% and 112.8%. Precision was expressed as relative standard deviation (RSD). The results of intraday and interday and for all analytes are listed in Table 5. The precision of the intraday RSDs was less than 6.3% and the precision of the interday RSDs was less than 8.5% in the fish samples. Typical MRM chromatograms of spiked samples are shown in Figure 2.

### 3.3. Analysis of Real Samples

In this work, the method applicability was checked by analyzing 30 fish samples collected from local markets. The samples originated from Heilongjiang province and Beijing city and were collected in May 2017 according to the procedures required by the surveillance program of the Chinese Ministry of Agriculture. The most prevalent compounds were sulfamethazine and enrofloxacin, and two samples surpassed the established MRL of 100 *μ*g kg^−1^ with values of 104 *μ*g kg^−1^ and 112 *μ*g kg^−1^ in fish samples. Ciprofloxacin was detected in almost all samples with high residual enrofloxacin samples because ciprofloxacin was a metabolite of enrofloxacin. The detected compounds were identified by means of retention time and ion ratio.  Table 6 summarizes the confirmatory and the quantitative analysis of the nine fish samples.

## 4. Conclusions

A selective and rapid UHPLC-MS/MS confirmatory method was developed and validated for the simultaneous detection of 22 SAs and 16 QNs, chloramphenicol, florfenicol, malachite green, and leucomalachite green in fish. The present work was to optimize sample extraction, purification, and chromatographic separation. The method enables the determination of the analyte residues in the low range and is qualified to carry out risk warnings for drug residues in fish.

## Figures and Tables

**Table 1:** Transitions and optimal conditions used for MS/MS analysis.

Analyte	Retention time (min)	Precursor ion(*m*/*z*)	Product ion(*m*/*z*)	Declustering potential (V)	Collision energy (eV)
Fleroxacin	7.73	370	269/326^*∗*^	90	26/19
Ofloxacin	7.75	362	261/318^*∗*^	85	27/18
Norfloxacin	7.75	320.3	233.4/276.3^*∗*^	85	26/26
Enoxacin	6.79	321	232/303^*∗*^	85	34/21
Ciprofloxacin	7.33	332.2	245.3/288.3^*∗*^	85	35/25
Enrofloxacin	8.07	360.2	245.2/316.3^*∗*^	90	39/27
Lomefloxacin	7.66	352	265/308^*∗*^	85	22/16
Danofloxacin	7.86	358	283/340^*∗*^	85	22/22
Orbifloxacin	7.96	396	295/352^*∗*^	85	24/17
Difloxacin	8.07	400	299/356^*∗*^	85	28/19
Sarafloxacin	8.05	386	299/342^*∗*^	85	26/18
Sparfloxacin	9.04	393	292/349^*∗*^	85	25/19
Oxolinic acid	9.72	262	216/244^*∗*^	85	29/18
Flumequine	10.07	262	202/244^*∗*^	85	32/18
Pefloxacin	7.20	334	290/316^*∗*^	85	18/20
Nalidixic acid	10.01	233.3	187.4/215.2^*∗*^	85	36/24
Sulfisoxazole	9.49	268	108/156^*∗*^	85	22/13
Sulfisomidine	7.05	279	186/156^*∗*^	85	17/19
Sulfathiazole	4.65	256	108/156^*∗*^	85	22/16
Sulfapyridine	4.98	250	184/156^*∗*^	85	18/16
Sulfamonomethoxine	8.44	281	215/156^*∗*^	85	17/17
Fleroxacin	7.49	281	215/156^*∗*^	85	17/17
Ofloxacin	6.90	254	108/156^*∗*^	85	22/16
Norfloxacin	7.21	271	107/156^*∗*^	85	30/14
Enoxacin	3.26	279	186/156^*∗*^	85	17/19
Ciprofloxacin	7.02	281	215/156^*∗*^	85	17/17
Enrofloxacin	5.50	265	172/156^*∗*^	85	17/17
Lomefloxacin	0.94	215	108/156^*∗*^	85	22/13
Danofloxacin	9.78	311	108/156^*∗*^	85	29/19
Orbifloxacin	9.28	311	108/156^*∗*^	85	29/19
Difloxacin	4.59	251	108/156^*∗*^	85	25/16
Sarafloxacin	8.34	285	108/156^*∗*^	85	24/15
Sparfloxacin	8.98	301	108/156^*∗*^	85	25/17
Oxolinic acid	8.84	277.3	107.7/156^*∗*^	80	20/12
Flumequine	8.90	285	108/156^*∗*^	80	37/24
Pefloxacin	11.05	315	160/158^*∗*^	80	23/25
Nalidixic acid	2.93	215	108/156^*∗*^	80	20/10
Sulfisoxazole	9.15	268	112.8/155.8^*∗*^	80	20/15
Sulfisomidine	9.90	329.3	208.1/313.1^*∗*^	110	49/49
Sulfathiazole	11.84	331.3	239/316^*∗*^	110	42/33
Sulfapyridine	10.55	321	257/152^*∗*^	-60	−16/−26
Sulfamonomethoxine	10.29	356	185/336^*∗*^	-60	−14/−27
Sulfadoxine-D3	9.29	314	156	85	17
Sulfadimethoxine-D6	9.78	317	156	85	20
Norfloxacin-D5	7.36	325.3	307.2	80	30
Ciprofloxacin-D8	7.57	340.3	322.3	85	29
Enrofloxacin-D5	8.24	365.3	321.3	94	28
Malachite green-D5	9.92	334.3	318.2	120	52
Leucomalachite green-D6	11.79	337.3	322.3	110	31
Chloramphenicol-D5	10.54	326	157	−60	−26

^*∗*^Transitions for quantification.

**Table 2:** Instrument conditions for target compounds analysis.

Ionization mode		ESI^ + ^ ESI^−^							
LC condition	Mobile phase	A	0.1% formic acid and 2 mM ammonium acetate						
		B	0.1% formic acid and acetonitrile						
	Gradient list	Time (min)	0.1	8	10	11	13	13.11	19
		A (%)	98	20	20	2	2	98	98
		B (%)	2	80	80	98	98	2	2
	Total flow	0.25 mL/min							
	Column temperature	40°C							
MS condition	Source temperature	550°C							
	Curtain gas	20 psi							
	Collision gas	Medium							
	Gas1	60 psi							
	Gas2	40 psi							
	Ion spray voltage	5000 v							

**Table 3:** The ratio of substrong fragment area to quantitative ion area.

Ion ratio of analytes (%)	Relative standard deviation (%)
>50	±20
20–50 (not including 20)	±25
10–20 (not including 10)	±30
≤10	±50

**Table 4:** Linearity for the determination of analytes in fish by liquid chromatography-tandem mass spectrometry.

Analyte	Standard equation	*R* ^2^	Standard addition equation^a^	*R* ^2^
Fleroxacin	*y* = 0.022*x* + 0.006	0.994	*y* = 0.060*x* + 0.053	0.991
Ofloxacin	*y* = 0.027*x* + 0.069	0.999	*y* = 0.075*x* + 0.011	0.999
Norfloxacin	*y* = 0.014*x* + 0.003	0.999	*y* = 0.047*x* + 0.002	0.998
Enoxacin	*y* = 0.077*x* + 0.349	0.998	*y* = 0.197*x* + 0.059	1.000
Ciprofloxacin	*y* = 0.017*x* + 0.129	0.994	*y* = 0.034*x* + 0.078	0.994
Enrofloxacin	*y* = 0.081*x* + 0.217	0.996	*y* = 0.030*x* + 0.038	0.999
Lomefloxacin	*y* = 0.010*x* + 0.034	0.991	*y* = 0.015*x* + 0.062	0.999
Danofloxacin	*y* = 0.024*x* + 0.037	0.999	*y* = 0.031*x* + 0.092	0.999
Orbifloxacin	*y* = 0.016*x* + 0.033	0.995	*y* = 0.007*x* + 0.069	0.998
Difloxacin	*y* = 0.012*x* + 0.021	0.994	*y* = 0.005*x* + 0.066	0.997
Sarafloxacin	*y* = 0.007*x* + 0.015	0.999	*y* = 0.003*x* + 0.022	0.999
Sparfloxacin	*y* = 0.015*x* + 0.035	0.999	*y* = 0.005*x* + 0.073	0.993
Oxolinic acid	*y* = 0.899*x* + 3.76	0.996	*y* = 0.501*x* + 0.034	0.992
Flumequine	*y* = 0.838*x* + 1.75	0.998	*y* = 0.359*x* + 0.022	0.998
Pefloxacin	*y* = 0.218*x* + 0.344	0.993	*y* = 0.445*x* + 0.027	0.999
Nalidixic acid	*y* = 1.18*x* + 6.58	0.993	*y* = 0.598*x* + 0.067	0.995
Sulfisoxazole	*y* = 0.014*x* + 0.048	0.992	*y* = 0.090*x* + 0.056	0.993
Sulfisomidine	*y* = 0.009*x* + 0.054	0.993	*y* = 0.007*x* + 0.091	0.992
Sulfathiazole	*y* = 0.023*x* + 0.075	0.995	*y* = 0.019*x* + 0.109	0.996
Sulfapyridine	*y* = 0.018*x* + 0.005	0.995	*y* = 0.063*x* + 0.322	0.999
Sulfamonomethoxine	*y* = 0.090*x* + 0.022	0.997	*y* = 0.082*x* + 0.181	0.998
Fleroxacin	*y* = 0.030*x* + 0.023	0.991	*y* = 0.022*x* + 0.306	0.994
Ofloxacin	*y* = 0.024*x* + 0.010	0.992	*y* = 0.013*x* + 0.032	0.999
Norfloxacin	*y* = 0.021*x* + 0.004	0.998	*y* = 0.022*x* + 0.231	1.000
Enoxacin	*y* = 0.009*x* + 0.014	0.997	*y* = 0.006*x* + 0.123	0.996
Ciprofloxacin	*y* = 0.009*x* + 0.065	0.996	*y* = 0.007 × 0.118	1.000
Enrofloxacin	*y* = 0.014*x* + 0.050	0.998	*y* = 0.012*x* + 0.124	0.992
Lomefloxacin	*y* = 0.003*x* + 0.015	0.999	*y* = 0.002*x* + 0.038	0.999
Danofloxacin	*y* = 0.046*x* + 0.099	0.993	*y* = 0.015*x* + 0.018	0.998
Orbifloxacin	*y* = 0.053*x* + 0.046	0.999	*y* = 0.039*x* + 0.097	0.999
Difloxacin	*y* = 0.011*x* + 0.052	1.000	*y* = 0.009*x* + 0.176	0.992
Sarafloxacin	*y* = 0.033*x* + 0.133	0.998	*y* = 0.018*x* + 0.083	0.997
Sparfloxacin	*y* = 0.006*x* + 0.043	0.999	*y* = 0.002*x* + 0.098	0.994
Oxolinic acid	*y* = 0.033*x* + 0.094	0.9988	*y* = 0.012*x* + 0.054	0.997
Flumequine	*y* = 0.002*x* + 0.001	0.999	*y* = 0.001*x* + 0.014	0.993
Pefloxacin	*y* = 0.017*x* + 0.033	1.000	*y* = 0.007*x* + 0.022	0.992
Nalidixic acid	*y* = 0.004*x* + 0.001	0.999	*y* = 0.004*x* + 0.028	0.993
Sulfisoxazole	*y* = 0.009*x* + 0.018	0.998	*y* = 0.004*x* + 0.127	0.993
Sulfisomidine	*y* = 0.614*x* + 0.085	0.995	*y* = 0.212*x* + 0.224	0.999
Sulfathiazole	*y* = 0.558*x* + 0.001	0.998	*y* = 0.191*x* + 0.104	0.997
Sulfapyridine	*y* = 0.338*x* + 0.024	0.999	*y* = 0.087 + 0.012	0.990
Sulfamonomethoxine	*y* = 0.324*x* + 0.012	1.000	*y* = 0.092*x* + 0.022	0.992

**Table 5:** Parameters of UPLC-MS/MS for the 42 compounds in fish sample.

Analyte	Spiked levels	Recoveries *R* (%)	Internal standard	LOD *μ*g·kg^−1^	LOQ *μ*g·kg^−1^	Intraday RSD(%) *μ*g·kg^−1^	Inter-day RSD(%) *μ*g·kg^−1^
Fleroxacin	0.5/1/4	71.2/73.4/75.5	Enrofloxacin-D5	0.1	0.4	3.6	4.1
Sulfamethoxazole	0.5/1/4	83.6/81.5/92.4	Sulfadimethoxine-D6	0.1	0.4	1.4	2.2
Ofloxacin	0.5/1/4	77.6/74.8/81.2	Enrofloxacin-D5	0.1	0.4	1.2	1.1
Sulfamethizol	0.5/1/4	74.4/78.8/82.5	Sulfadimethoxine-D6	0.1	0.4	2.7	3.3
Norfloxacin	1/2/10	101/102/106	Norfloxacin-D5	0.25	1.0	4.4	3.8
Sulfamethazine	0.5/1/4	92.2/95.4/96.7	Sulfadimethoxine-D6	0.1	0.4	1.5	1.7
Enoxacin	0.5/1/4	75.5/73.6/78.9	Enrofloxacin-D5	0.1	0.4	1.8	2.3
Sulfameter	0.5/1/4	88.1/90.3/94.6	Sulfadimethoxine-D6	0.1	0.4	1.8	2.5
Ciprofloxacin	1/2/10	82.1/85.4/88.6	Ciprofloxacin-D8	0.25	1.0	2.4	2.6
Sulfamerazine	0.5/1/4	95.5/94.6/1003	Sulfadimethoxine-D6	0.1	0.4	1.3	3.4
Enrofloxacin	0.5/1/4	101/104/107	Enrofloxacin-D5	0.1	0.4	2.7	1.2
Sulfaguanidine	0.5/1/4	71.6/72.4/76	Sulfadimethoxine-D6	0.1	0.4	1.3	2.3
Lomefloxacin	1/2/10	75.5/75/77.3	Enrofloxacin-D5	0.25	1.0	3.4	3.7
Sulfadoxine	0.5/1/4	105/107/104	Sulfadoxine-D3	0.1	0.4	2.1	4.3
Danofloxacin	0.5/1/4	76.6/76.7/81	Enrofloxacin-D5	0.1	0.4	1.6	1.9
Sulfadimethoxine	0.5/1/4	83.2/87.2/91.8	Sulfadimethoxine-D6	0.1	0.4	1.4	2.7
Orbifloxacin	0.5/1/4	83.6/83.8/87	Enrofloxacin-D5	0.1	0.4	1.1	1.8
Sulfadiazine	0.5/1/4	92.6/93.2/103	Sulfadoxine-D3	0.1	0.4	2.3	3.8
Difloxacin	0.5/1/4	72.2/77.5/77	Enrofloxacin-D5	0.1	0.4	1.5	1.4
Sulfachloropyridazine	0.5/1/4	82.6/82.7/84.2	Sulfadimethoxine-D6	0.1	0.4	2.2	4.5
Sarafloxacin	1/2/10	84.9/77.5/83.4	Sulfadimethoxine-D6	0.25	1.0	1.4	2.2
Sulfachinoxalin	0.5/1/4	67.2/71/71.5	Sulfadimethoxine-D6	0.1	0.4	2.6	4.1
Sparfloxacin	1/2/10	84.4/86.5/78.3	Enrofloxacin-D5	0.25	1.0	1.8	2.5
Sulfabenzamide	0.5/1/4	71.6/72.4/75.8	Sulfadimethoxine-D6	0.1	0.4	3.1	5.6
Oxolinic acid	0.5/1/4	69.2/71.4/73.3	Enrofloxacin-D5	0.1	0.4	2.7	3.2
Sulfachloropyrazine	2001/2/10	73.7/81/76.3	Sulfadimethoxine-D6	0.25	1.0	2.4	3.5
Flumequine	0.5/1/4	68.2/72.2/77.5	Enrofloxacin-D5	0.1	0.4	1.7	1.6
Sulfaphenazole	0.5/1/4	70.4/72.3/73.8	Sulfadimethoxine-D6	0.1	0.4	3.5	2.8
Pefloxacin	0.5/1/4	80.2/79/82.4	Enrofloxacin-D5	0.1	0.4	3.5	3.8
Sulfacetamide	0.5/1/4	74.2/75/80	Sulfadimethoxine-D6	0.1	0.4	3.7	3.5
Nalidixic acid	0.5/1/4	69.6/69.8/73.5	Enrofloxacin-D5	0.1	0.4	5.2	6.7
Sulfamoxol	0.5/1/4	83.6/85.2/87.8	Sulfadimethoxine-D6	0.1	0.4	3.3	4.6
Sulfisoxazole	1/2/10	93.5/93/89.8	Sulfadimethoxine-D6	0.25	1.0	1.1	2.4
Sulfamethoxypyridazine	0.5/1/4	78/81.9/86.2	Sulfadimethoxine-D6	0.1	0.4	1.7	1.8
Sulfisomidine	1/2/10	73.4/77/82.2	Sulfadimethoxine-D6	0.25	1.0	2.5	2.2
Sulfathiazole	0.5/1/4	83.8/80.7/81.5	Sulfadimethoxine-D6	0.1	0.4	2.6	3.3
Sulfapyridine	0.5/1/4	67/69.8/72.5	Sulfadimethoxine-D6	0.1	0.4	4.4	7.1
Sulfamonomethoxine	0.5/1/4	87/89.4/92	Sulfadimethoxine-D6	0.1	0.4	1.2	1.9
Malachite green	0.5/1/4	91/93.3/92.2	Malachite green-D5	0.1	0.4	2.6	1.6
Leucomalachite green	0.5/1/4	86.5/91.9/92.8	Leucomalachite green-D6	0.1	0.4	1.9	4.2
Chloramphenicol	0.8/4.0/20	91.6/95.2/106	Chloramphenicol-D5	0.15	0.5	1.6	3.8
Florfenicol	0.8/4.0/20	87.9/92.3/98	Chloramphenicol-D5	0.15	0.5	2.1	1.7

**Table 6:** Confirmatory and quantitative analysis of samples.

Analytes	Tissue	Residue amount (*μ*g·kg^−1^)	Ratio of analyte in sample (%)	Ratio of analyte in standard (%)
Sulfamethazine	Fish sample-1	41.3	30.2	37.1
	Fish sample-2	31.5	33.4	
	Fish sample-3	104	35.1	

Enrofloxacin	Fish sample-4	65.1	42.2	48.8
	Fish sample-5	14.8	40.3	
	Fish sample-6	15.5	44.6	
	Fish sample-7	73.6	51.5	
	Fish sample-8	45.5	57.7	
	Fish sample-9	112	53.6	

Ciprofloxacin	Fish sample-4	14.6	52.2	53.7
	Fish sample-7	16.3	55.6	

## Data Availability

The data used to support the findings of this study are available from the corresponding author upon request.
